# Effects of Different Container Types on (1→3)-β-D-glucan Recovery

**DOI:** 10.3390/molecules28196931

**Published:** 2023-10-04

**Authors:** Luisa Burgmaier, Bernhard Illes, Michael Leiss, Meltem Avci-Adali, Johannes Reich

**Affiliations:** 1 Microcoat Biotechnologie GmbH, Am Neuland 3, 82347 Bernried am Starnberger See, Germany; l.burgmaier@microcoat.de (L.B.); b.illes@microcoat.de (B.I.); 2 Clinical Research Laboratory, Department of Thoracic, Cardiac and Vascular Surgery, University Hospital Tuebingen, Tuebingen University, Calwerstr. 7/1, 72076 Tuebingen, Germany; meltem.avci-adali@uni-tuebingen.de; 3 Roche Diagnostics GmbH, Nonnenwald 2, 82377 Penzberg, Germany

**Keywords:** (1→3)-β-D-glucan, container effect, sample hold time, SHT, freeze/thaw, spike recovery

## Abstract

It has long been known that containers for sample analysis or storage can play a role in endotoxin recovery and have to be taken into account when determining endotoxin concentrations. However, there is little data on the effects of containers regarding (1→3)-β-D-glucan, which plays a role as a contaminant in endotoxin measurements. To determine the effect of the container on (1→3)-β-D-glucan measurements, four different types of containers were investigated at different temperatures and stored for up to 28 days. For short-term storage for 3 h at room temperature, no effect of the container on the (1→3)-β-D-glucan recovery could be observed, but for storage at −20 °C, the results indicate that the storage time and temperature influences (1→3)-β-D-glucan detection. All containers showed a trend of lower recoveries over time, but the polyethylene container showed a significantly lower recovery compared to the other containers. We also showed that freeze/thaw cycles had a strong influence on the recovery of (1→3)-β-D-glucan in polyethylene containers. Our study showed that the container can affect not only the detection of endotoxins but also the detection of (1→3)-β-D-glucans.

## 1. Introduction

The contamination of pharmaceutical products with glucans can occur within a number of upstream and downstream processes, such as filtration during purification. The potential origins are cellulose-based materials (e.g., cellulose filters) or fungal contamination of raw materials (e.g., sucrose-containing buffers) [1,2]. (1→3)-β-D-glucans are polysaccharides composed of glucose monomers that can trigger an inflammatory response, similar to endotoxins. They are produced by most fungi as a cell wall component, but they are also found in a wide range of other eukaryotic and prokaryotic organisms, like yeast and algae. They are also a component of plant tissue. They possess high structural diversity, e.g., differences in polymer length. This also results in different properties regarding biological activity and solubility. The least water-soluble particulate forms are associated with more potent immunological activity [3,4].

The detection of (1→3)-β-D-glucans employs a similar enzymatic cascade to the detection of endotoxins. Both substances trigger the Limulus Amoebocyte Lysate (LAL) reaction, utilizing blood cells (amoebocytes) sourced from horseshoe crabs. In the cascade mediated by LPS, three distinct serine protease zymogens are involved: factor C, factor B, and proclotting enzyme, in addition to coagulogen, a protein possessing the capacity to form gel-like structures. This cascade commences with LPS catalyzing the conversion of zymogen factor C into the active factor C_a_. Subsequently, factor C_a_ activates factor B, leading to the conversion of proclotting enzyme into clotting enzyme. The clotting enzyme then cleaves two peptide bonds in coagulogen, a molecule resembling fibrinogen, to form insoluble coagulin gel. The above-described cascade can also be initiated by (1→3)-β-D-glucan. Initially, factor G, a zymogen of serine protease, undergoes activation and subsequently triggers the activation of the proclotting enzyme [5]. As a consequence, employing the LAL assay for endotoxin measurement may yield false-positive results in the presence of (1→3)-β-D-glucans [6]. Fortunately, solutions are available to address this interference issue. To mitigate such interference, the test can be altered to include glucan-inhibiting substances [7], or an alternative approach involving the use of a recombinant factor C assay can be used. In the recombinant factor C (rFC) assay, factor G is absent, preventing the cascade’s activation by this protein in response to (1→3)-β-D-glucans. rFC-based testing methods have proven to be reliable and, notably, exhibit greater specificity than LAL tests [8]. For glucan detection, factor C is removed from the lysate, allowing the remaining factor G to initiate the cascade upon encountering (1→3)-β-D-glucans [9].

In comparison with bacterial endotoxins, (1→3)-β-D-glucans exhibit milder immunological activity but retain immunomodulatory characteristics. They are recognized for their ability to bind to and stimulate various cell types, such as monocytes and macrophages [10,11,12]. Furthermore, it has been observed that (1→3)-β-D-glucans can augment the detrimental effects of endotoxin-induced toxicity, thereby impacting the immune system adversely [13]. Unlike bacterial endotoxins, there is no universally accepted standard for detecting (1→3)-β-D-glucans. Nevertheless, there is an emerging pattern in the industry and among global regulatory bodies to recognize and quantify (1→3)-β-D-glucan impurities and to comprehend the factors that affect their detection [1,14,15].

One often-overlooked aspect of contaminant detection is the effect of the container. It has long been established that, besides the low endotoxin recovery phenomenon [16,17], the container (e.g., adsorption) can also influence endotoxin determination. Different types of plastic lead to differences in endotoxin recovery and even the initial endotoxin concentration or source of the endotoxin can lead to differences in recovery [18]. Another factor that can influence endotoxin recovery is the storage time of the samples in a specific container, with containers made of certain materials like polycarbonate showing decreasing recoveries over time. This effect of endotoxin adsorption to the surface of containers is more strongly pronounced in plastic containers than in those made of borosilicate [19]. However, this effect can also be influenced by the samples, leading to differences in adsorption in the presence of different materials or the charge of the samples [20]. Taking all of these factors into account, borosilicate containers are the preferred containers for performing endotoxin testing and studies.

However, little is known about how differences in container properties affect the recovery of (1→3)-β-D-glucan. As (1→3)-β-D-glucan contamination is often accompanied by endotoxin contamination, it is important that accurate measurements can also be performed for (1→3)-β-D-glucan, regardless of the container type, to obtain an accurate picture of the total contamination.

This study investigated the recovery of (1→3)-β-D-glucan from containers composed of borosilicate, polypropylene, or polyethylene for short-term storage at room temperature (RT; 18–25 °C) and long-term storage at −20 °C (−30–−10 °C). This study aimed to investigate the temperature, time, and container dependencies of (1→3)-β-D-glucan recovery and to provide recommendations for the best practice for obtaining the most accurate results.

## 2. Results and Discussion

In the following, we present our findings regarding the contamination of different containers with (1→3)-β-D-glucan and the influence of storage under different conditions. First, a short-term storage at RT was performed, which mimicked the processing time between sampling and possible analyses, such as immediate in-process controls. Subsequently, a sample-hold time study was performed at −20 °C to mimic the storage of, e.g., retain samples. In addition, the influence of freezing and thawing was investigated.

### 2.1. Initial Contamination of Containers

First, before the investigation of the effects of the container on the recovery of (1→3)-β-D-glucan, the contamination of different container types was determined. For this, 10 containers of each type were filled with 1 mL reagent-grade water (RGW) heated to 37 °C and vortexed for 15 s. The containers were incubated for 60 min at RT, and after 30 min or 60 min, the test containers were vortexed again for 15 s. The (1→3)-β-D-glucan content was measured without additional dilution.

The results of these investigations are summarized in Table 1. All 10 individual containers tested per material showed no initial contamination above the limit of detection (6.26 pg/mL) with (1→3)-β-D-glucan. The positive product controls (PPC), which ensured that no test interference occurred, were valid for all samples.

Labware is often labelled as sterile, RNAse-free, DNAse-free, or pyrogen-free. Sterile defines a product as absent of any living organisms; however, nonviable biologic contaminations may not be removed via standard sterilization processes. RNAse- and DNAse-free defines, as stated, a product that is free of RNAse or DNAse. Endotoxin-based contaminations could be excluded under the pyrogen-free label [21]. None of these labels guarantee the exclusion of glucan contaminations. It is, therefore, important to exclude possible contaminations prior to sampling. Our data showed glucan contamination below the detection limit for all tested containers, so the investigated containers were suitable for glucan sample collection.

### 2.2. Short-Term Storage at Room Temperature

For the investigation of the effects of short-term storage on the recovery of (1→3)-β-D-glucan, four different containers were filled with 1 mL (1→3)-β-D-glucan-spiked RGW to imitate a glucan contamination. The initial spike concentration was set at 25 pg/mL. All samples were measured directly after adding the spiked sample and after an incubation time of 3 h or 24 h at RT. As a confirmation of the spike concentration, a water control was prepared using the same spike solution.

**Figure 1:**
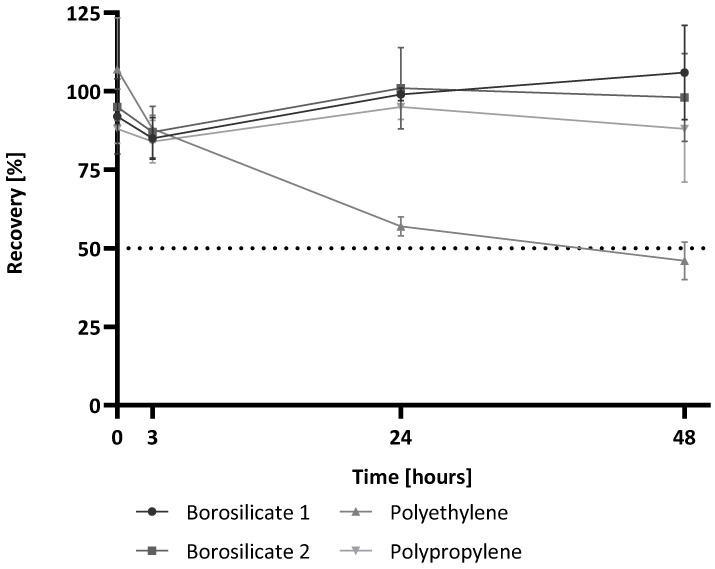
(1→3)-β-D-glucan recovery after the short-term storage of spiked reagent-grade water in four types of containers at room temperature for up to 48 h. (1→3)-β-D-glucan recovery for each time point is shown. Time point zero (0 h) of the water control with the 25 pg/mL (1→3)-β-D-glucan spike was used as the reference value and set to 100% for calculations. The recovery is shown relative to this reference value. Each point shows the mean recovery of three replicates. The error bars reflect the standard deviation of replicates (n = 3). The dotted line marks 50% recovery.

The results of the short-time storage study are shown in Figure 1. For the measurement of the samples at time point (Tmp) 0 h, no significant differences between the different containers are observed. All containers reach a recovery above 80% calculated to the water control at Tmp 0 h (borosilicate 1: 92%, borosilicate 2: 88%, polyethylene: 107%, polypropylene: 95%). The measurement after storage of the samples for 3 h at RT also showed valid recovery of the glucan spike (85%, 84%, 88%, 87%). Even after 24 h and 48 h of storage, all samples show stable (1→3)-β-D-glucan recoveries around 100%, except for the sample in the polyethylene container. The recovery in this sample is only 57% after 24 h and even sinks below the 50% limit after 48 h (46%). Thus, it shows a significant decrease compared to the water control at Tmp 0 h.

Regardless of the differences in material or surface-area-to-volume ratios, all containers appeared to be well suited for glucan detection after short-term storage for 3 h. If samples are stored for longer than 3 h, effects appear with the use of polyethylene containers. The use of polyethylene containers poses the risk of underestimating potential contamination. These results are in contrast to the container effects observed for endotoxin analysis. Meadows et al. showed that, up to 24 h, there was no significant difference in endotoxin recovery in different containers of different materials and surface-area-to-volume ratios (polystyrene tube, polypropylene tube, glass tube) [19].

### 2.3. Long-Term Storage at −20 °C

**Figure 2:**
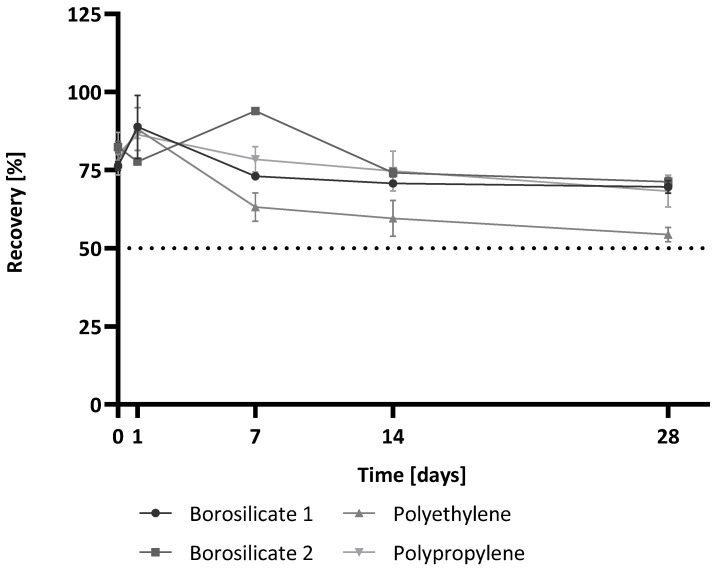
(1→3)-β-D-glucan recovery after the long-term storage of spiked reagent-grade water in four types of containers at −20 °C for up to 28 days. (1→3)-β-D-glucan recovery for each time point is shown. Time point zero (0 h) of the water control with the 25 pg/mL (1→3)-β-D-glucan spike was used as the reference value and set to 100% for calculations. The recovery is shown relative to this reference value. Each point shows the mean recovery of three replicates. The error bars reflect the standard deviation of replicates (n = 3). The dotted line marks 50% recovery.

As the short-term storage of samples in the containers did, in most cases, not correspond to the storage conditions used in industry, longer storage times at −20 °C were also investigated. It was also hoped that freezing the samples might reduce or prevent the observed container effects. Sample preparation was identical to that of the short-term storage experiments. Samples were stored at −20 °C and thawed for measuring after specific storage times (0 h, 1 d, 7 d, 14 d, 28 d). The recoveries at each time point for each container type are shown in Figure 2.

For time point 0 h, all container types showed comparable recoveries. This was in common with the results for time point Tmp 0 h of the short-term storage study. After 1 d of storage and thawing, all of the containers showed valid recoveries in a similar range (borosilicate 1: 89%, borosilicate 2: 86%, polyethylene: 88%, polypropylene: 78%). The mean recoveries over all time points were: for borosilicate 1, 76% with a coefficient of variation (CV) of 10%; for polypropylene, 77% (CV 10%); for polyethylene, 68% (CV 21%); and for borosilicate 2, 80% (CV 11%). It was notable that for the polyethylene container, the mean recovery was the lowest and also showed the highest variance.

These results indicate that the storage time influences glucan detection regardless of the container type. However, the container material also appears to have an effect. Although all containers show a trend of lower recoveries after storage at −20 °C and all recoveries remain above 50% of the water control at Tmp 0 h for all time points, the use of polyethylene containers again has the risk of underestimating a potential contamination. However, the data show that the container effects of polyethylene occur much later when stored at −20 °C than at RT. This could, therefore, indicate an energy-dependent process.

### 2.4. Freeze and Thaw Comparison

**Figure 3:**
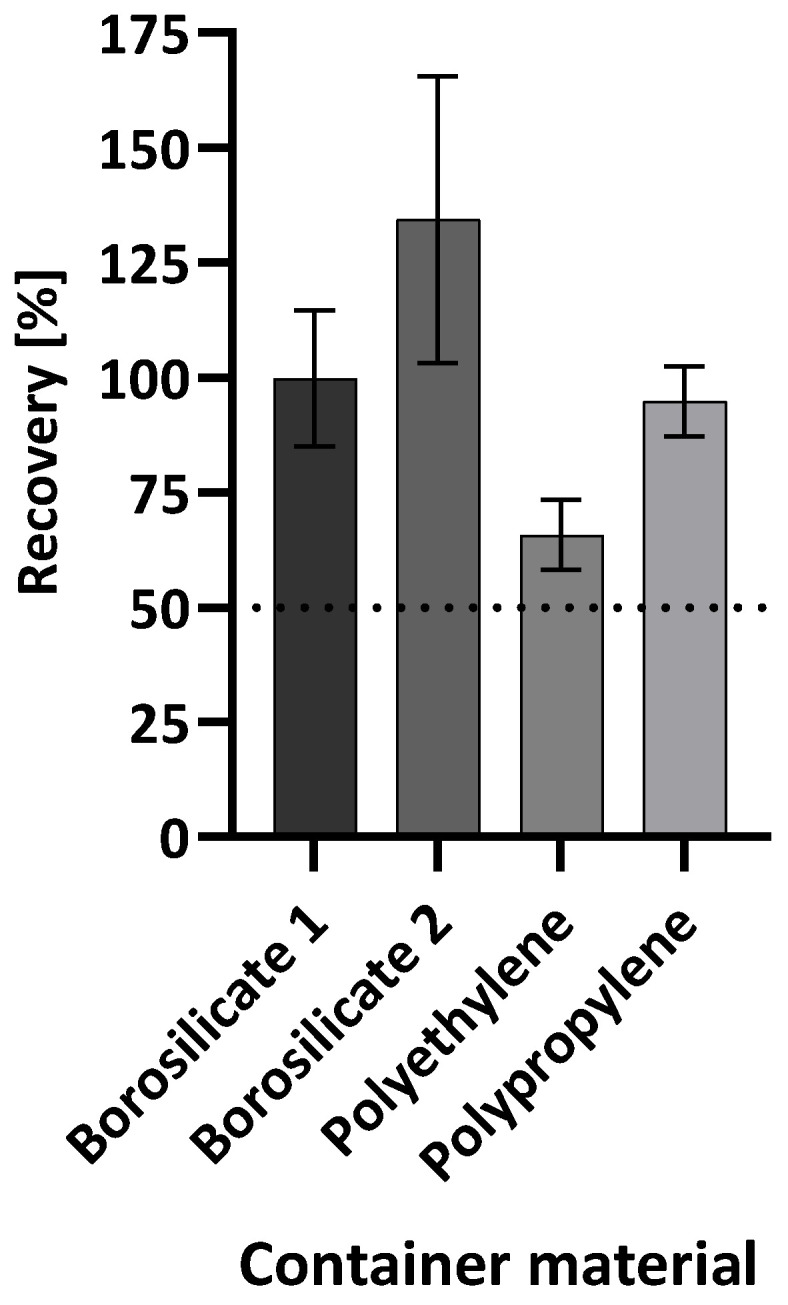
Mean (1→3)-β-D-glucan recovery after one freeze/thaw cycle of spiked reagent-grade water in four types of containers. A water control with a 25 pg/mL (1→3)-β-D-glucan spike that was not subjected to the freeze/thaw cycle was used as the reference value and set to 100% for calculations. The recovery is shown relative to this reference value. Each bar shows the mean recovery of three replicates. The error bars reflect the standard deviation of replicates (n = 3). The dotted line marks 50% recovery.

To investigate whether a freeze-and-thaw process also has an influence on recovery depending on the container type, a further experiment was performed. Water spiked with (1→3)-β-D-glucan was used to fill each of the containers and subjected to a freeze/thaw cycle with 15 min thawing time before measuring. As a reference, water spiked with (1→3)-β-D-glucan was used to fill each container and was not subjected to the freeze/thaw cycle. As can be seen in Figure 3, all samples in the different containers reached recoveries over 50% of the control. In the borosilicate 1, borosilicate 2, and polypropylene containers, recoveries of 100%, 134%, and 95%, respectively, were determined. Only for the polyethylene container was the recovery lower (66%). This indicates that the freeze/thaw cycle also influences the recovery of (1→3)-β-D-glucan in polyethylene containers.

The results indicate that the freeze/thaw cycle generally has no effect on the recovery of beta-glucan. For three of the four containers, the recovery rate was close to 100% of the respective control without freezing. For borosilicate 1, borosilicate 2, and polypropylene, storage time and storage temperature appear to have a greater impact on glucan detection than freeze/thaw cycles.

**Figure 4:**
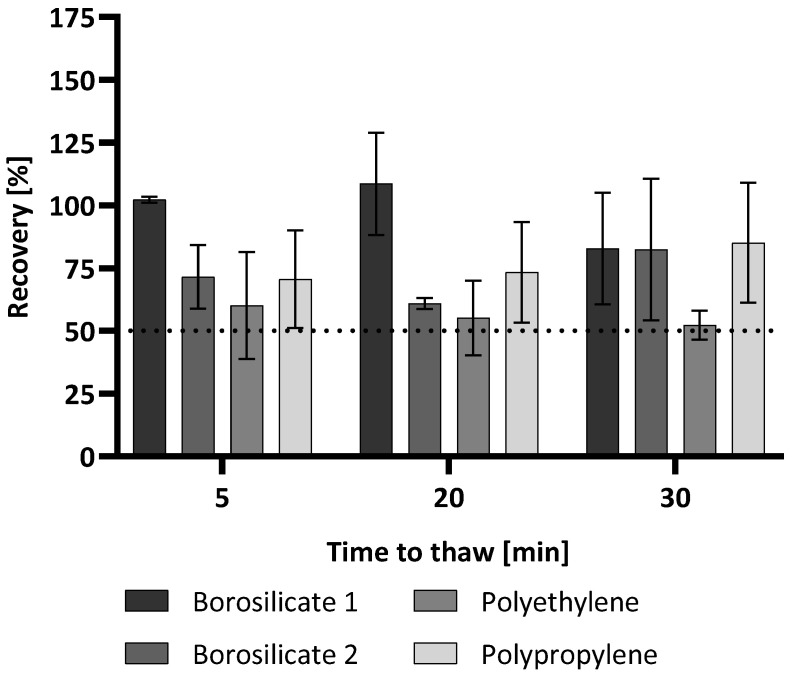
(1→3)-β-D-glucan recovery after one freeze/thaw cycle with different thawing times in four container types. (1→3)-β-D-glucan recovery for each thawing time is shown. A water control with a 25 pg/mL (1→3)-β-D-glucan spike that was not subjected to the freeze/thaw cycle was used as the reference value and set to 100% for calculations. The recovery is shown relative to this reference value. Each bar shows the mean recovery of two replicates. The error bars reflect the standard deviation of replicates (n = 2). The dotted line marks 50% recovery.

To further investigate the effect of thawing times and possible container dependencies, the recovery of (1→3)-β-D-glucan was investigated after 5, 20, and 30 min of thawing. The overall process was handled as before where a 15 min thawing time was used before measuring. The only difference was the time the samples were allowed to thaw and acclimate before measuring. After thawing times of 5 min and 20 min, the recoveries for all containers aside from borosilicate 1 were below 75% compared to the control, which was not subjected to the freeze/thaw cycle. While the recoveries for the samples in the borosilicate 2 and polypropylene containers increased with a longer thawing time (30 min), the polyethylene container showed an even lower recovery. This was only slightly above 50% (52%) (Figure 4).

### 2.5. Dependence of Surface-Area-to-Volume Ratio

**Figure 5:**
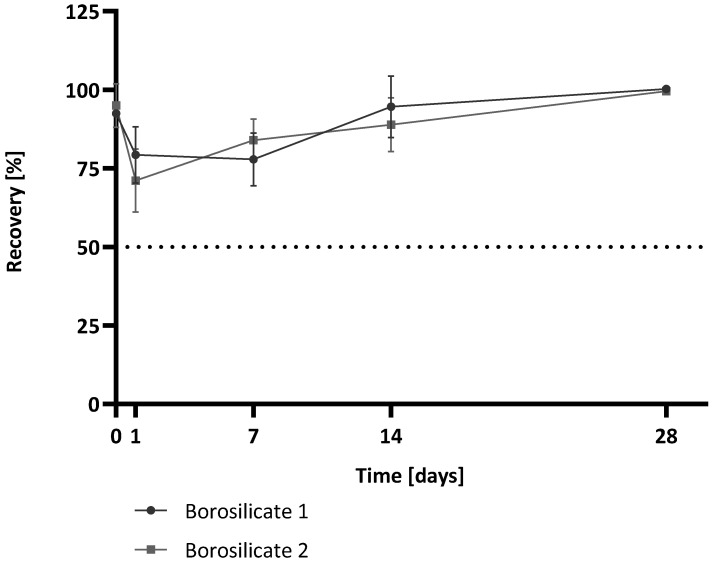
(1→3)-β-D-glucan recovery after the long-term storage of spiked reagent-grade water in two types of borosilicate containers at 4 °C for up to 28 days. (1→3)-β-D-glucan recovery for each time point is shown. Time point zero (0 h) of the water control with the 25 pg/mL (1→3)-β-D-glucan spike was used as the reference value and set to 100% for calculations. The recovery is shown relative to this reference value. Each bar shows the mean recovery of three replicates. The error bars reflect the standard deviation of replicates (n = 3). The dotted line marks 50% recovery.

In a further experiment, the influence of the surface-area-to-volume ratio was investigated. For this study, only the two borosilicate containers were compared, as they differed only in their surface-area-to-volume ratio and were, therefore, best suited for comparison. For this purpose, samples were kept in the containers at 4 °C for 28 days. In order to avoid freeze/thaw effects, storage at 4 °C was chosen. This is also in common with the storage of samples in industry when a sample cannot be immediately analysed. Sample preparation was performed according to the −20 °C long-time storage experiments. Samples were stored at 4 °C and measured directly after adding the spiked sample and after a specific storage time (0 h, 1 d, 7 d, 14 d, 28 d). The recoveries at each time point for the two borosilicate containers are shown in Figure 5.

Both containers show similar behaviours over the tested time interval. For time point 0 h, both containers showed recoveries near 100% (92% for borosilicate 1, 95% for borosilicate 2). After 1 d of storage, the recoveries are slightly lower for both sample containers compared to Tmp 0 h (79% for borosilicate 1, 71% for borosilicate 2). Nevertheless, both container types revealed recoveries of 100% after storage for 28 d. The mean recoveries over all time points were: for borosilicate 1, 89% (CV 11%), and for borosilicate 2, 88% (CV 13%). Although both containers have different surface-area-to-volume ratios, the recoveries are almost identical.

The surface-area-to-volume ratio plays an important role in many biological processes and influences, for example, pharmacokinetic and pharmacodynamic parameters [22]. Also, in assay development, the surface/volume ratio is a considering factor. In solid phase assays like ELISA (enzyme-linked immunosorbent assay), a larger surface-area-to-volume ratio implies a faster adsorption of molecules from the liquid phase because the liquid is exposed to more surface [23]. Therefore, it is conceivable that a container with a larger surface-area-to-volume ratio has a greater impact on (1→3)-β-D-glucan recovery than a container with a smaller surface-area-to-volume ratio. The sample and possible (1→3)-β-D-glucan contamination are more exposed to the material. However, our data indicate that borosilicate as a container material does not influence the recovery when stored at 4 °C over 28 d and, therefore, the surface-area-to-volume ratio does not influence recovery.

## 3. Materials and Methods

### 3.1. Materials and Reagents

(1→3)-β-D-glucan standard, lyophilized Glucatell reagent, and RGW were purchased from Associates of Cape Cod (East Falmouth, MA, USA). The used container types and their suppliers are summarized in Table 2.

### 3.2. Beta-Glucan Assay

The (1→3)-β-D-glucan concentrations were determined using the Glucatell assay kit following the manufacturer’s instructions (Associates of Cape Cod). The detection is based on a modification of the Limulus Amoebocyte Lysate (LAL) pathway and therefore specific for (1→3)-β-D-glucan. As procedure, the kinetic mode using onset O.D. was chosen, with a log—log plot of the onset times versus the standard concentrations. The glucan standard was reconstituted with RGW to provide a solution of 100 pg glucan/mL. The standard curve was prepared via serial dilution of the stock solution (100 pg/mL–3.125 pg/mL). A total of 25 µL of the standard or sample was added to each well of the microtiter plate. The Glucatell reagent was reconstituted with RGW and Pyrosol buffer, and then 100 µL of the reagent was added to each well of the plate. The Glucatell lysate/sample mixture was incubated at 37 °C ± 1 °C and measured using an ELx808 Absorbance Microplate Reader (BioTek, Bad Friedrichshall, Germany) equipped with a 405 nm filter. Analysis was executed using the Gen5 Secure software, version 3.11 (BioTek, Bad Friedrichshall, Germany). All samples were tested in duplicate. The glucan concentration was determined from the mean of the readings interpolated from the kinetic standard curve. Samples were accepted as valid if the CV of the duplicates was less than 30%.

### 3.3. Calculation of Recovery Rate

The determined (1→3)-β-D-glucan content in the tested samples was determined relative to the total content at time point zero in water controls and stated as a percent. Water controls were prepared by spiking (1→3)-β-D-glucan in RGW using a 25 pg/mL (1→3)-β-D-glucan spike solution.

For the freezing/thawing experiments, the content of (1→3)-β-D-glucan in the tested sample was determined and the recovery rate was determined relative to the content in the water spiked with a 25 pg/mL (1→3)-β-D-glucan spike solution that was used to fill each container that was not subjected to the freeze/thaw cycle.

The obtained results were rated as valid when the recovery of the (1→3)-β-D-glucan content was between 50% and 200%.

The calculation of the mean and standard deviation as well as the creation of figures were performed using Graph Pad Prism, version 10.0.0.

## 4. Conclusions and Future Directions

The growing scientific evidence of the immunomodulatory effects of beta-glucans results in an increasing need for its reliable quantification. While there are established detection methods, little is known about the potential influences on beta-glucan analysis and more work needs to be performed to validate these methods and to establish compendial procedures for their application [24,25,26,27].

It is known that endotoxin recovery depends on various parameters, including but not limited to the plastic resin manufacturer, sample container manufacturer, and endotoxin species. However, less is known about the influence of these parameters on (1→3)-β-D-glucan.

Our study shows that the container can also have an influence on the detection of (1→3)-β-D-glucans. The material and storage time as well as storage temperature play important roles. In this study, four different container types for storage at different temperatures and time intervals were analysed. The data showed that the polypropylene and borosilicate containers had the most reliable results over time. The standard deviation of the average of glucan recovery across all time points in the long-term study was 7% for borosilicate 1, 6% for borosilicate 2, and 8% for polypropylene compared to 13% for the polyethylene container. Consequently, sampling containers should be selected with care. In general, all tested containers were suitable for short-term storage, as no initial contamination of the containers could be detected. When the measurement is performed within 3 h of the sampling event, all containers can be recommended. If samples for the detection of (1→3)-β-D-glucans are subjected to storage for more than 3 h in the container, independent of storage temperature, polyethylene is not recommended as a container material. Borosilicate glass as well as polypropylene containers appear to be equally suitable for the detection of (1→3)-β-D-glucans. Although polypropylene is chemically quite similar to polyethylene, it is much harder, stronger, and more thermally resilient. These differences could influence the adsorption properties of the materials [28].

The long-term storage study was conducted at only one temperature (−20 °C), as this is the most common storage temperature for samples for long-term storage in industry. However, it would be interesting to observe how other temperatures (4 °C, −80 °C) affect (1→3)-β-D-glucan detection in all four containers used in this study. This will be the subject of further studies.

Another point that should not be overlooked is the surface-area-to-volume ratios of the containers. It is possible that this also plays a role in the observed effects on the recoveries as the polyethylene container had the lowest volume of the investigated containers. The comparison of both borosilicate containers, which possess different surface-area-to-volume ratios, has shown that during long-term storage at 4 °C the surface/volume ratio has no influence on the recovery of (1→3)-β-D-glucan, although a dependence on the surface/volume ratio cannot be completely excluded with the investigations performed. The effects of surface-area-to-volume ratios of other container materials should be further investigated in follow-up studies. It should be noted that in this initial investigation, only water spiked with a standard (1→3)-β-D-glucan was used. Thus, it would be interesting to know if the sample matrix or glucan species also contribute to this container effect. This should be part of further research.

## Figures and Tables

**Table 1:** Initial contamination of containers used in this study.

Container	Average (1→3)-β-D-glucan Concentration [pg/mL]	Average PPC	Number of Valid Measurements
Borosilicate 1	<6.26	109	10
Borosilicate 2	<6.26	134	9 (1 CV > 30%)
Polyethylene	<6.26	113	10
Polypropylene	<6.26	96	10

**Table 2:** Container types used in this study.

Container	Material	Volume	Supplier
PyroControl	Borosilicate glass (1)	10 mL	ACILA AG (Mörfelden-Walldorf, German)
EndoGrade Glass Test Tubes	Borosilicate glass (2)	6 mL	BioMerieux (Marcy-l’Étoile, France)
Nalgene Cryoware	Polyethylene	2 mL	Thermo Fisher Scientific (Waltham, MA, USA)
CELLSTAR Tubes	Polypropylene	15 mL	Greiner bio-one GmbH (Frickenhausen, Germany)

## Data Availability

Not applicable.
